# Evaluating the Accuracy of Remote Questionnaire and Speech Measures in Detecting Cognitive Impairment in the ADNI4 Remote Digital Cohort: Cross-Sectional Study

**DOI:** 10.2196/108372

**Published:** 2026-11-06

**Authors:** Rayan M Ashish, Jessica A Turner

**Affiliations:** 1 Head-Royce School, 4315 Lincoln Avenue, Oakland, CA, 94602, United States, 1 510-228-1592; 2 Department of Psychiatry and Behavioral Health, Wexner Medical Center, The Ohio State University, Columbus, OH, United States

**Keywords:** subjective cognitive decline, Everyday Cognition scale, 12-item Everyday Cognition, ECog-12, mild cognitive impairment, Alzheimer disease, dementia, speech analysis, digital biomarkers, remote monitoring, digital health

## Abstract

**Background:**

Scalable remote approaches are increasingly needed to identify cognitive impairment in clinical research. The Everyday Cognition (ECog) scale and its short form (12-item Everyday Cognition [ECog12]) capture subjective cognitive and functional decline, whereas automated speech-based story recall provides an objective measure of cognitive performance. Whether speech recall contributes information beyond remote questionnaire assessment remains unclear.

**Objective:**

This study aimed to determine whether automated speech-based story recall measures provide incremental discrimination of cognitive impairment diagnoses beyond the self-reported ECog12 (Self-ECog12) scale and demographics, both without and with 2 additional subjective memory measures that assess current memory/thinking concerns and perceived recent decline.

**Methods:**

This cross-sectional study included 7268 individuals from the Alzheimer’s Disease Neuroimaging Initiative 4 (ADNI4) Remote Digital cohort with complete baseline questionnaire, demographic, and Storyteller speech-recall data. The outcome was defined as a participant report of a prior diagnosis of mild cognitive impairment or memory problem, Alzheimer disease, or dementia (n=574, 7.9%). Participants completed a smartphone-based Storyteller assessment involving immediate and delayed spoken recall of standardized stories; recordings were automatically analyzed to derive 5 prespecified recall and language measures. Participants were stratified into training and independent natural-prevalence test cohorts. Logistic regression models evaluated Self-ECog12 alone; Self-ECog12 plus demographics; Storyteller added to that model; the 2 additional subjective memory measures; and the full model incorporating Storyteller. Model development and threshold selection were confined to training data. Discrimination, calibration, and threshold-dependent performance were evaluated in the held-out test cohort. Area under the receiver operating characteristic curve (AUC) comparisons used paired DeLong tests.

**Results:**

The test cohort included 1817 participants, of whom 143 (7.9%) reported a prior diagnosis. Self-ECog12 alone achieved an AUC of 0.792 (95% CI 0.755-0.828), increasing to 0.827 (95% CI 0.792-0.859) with demographics. Adding Storyteller increased AUC to 0.853 (95% CI 0.821-0.883; ΔAUC 0.026, 95% CI 0.009‐0.043; paired DeLong *P*=.002). With the 2 subjective memory measures included, AUC was 0.857 (95% CI 0.830-0.882) and increased to 0.875 (95% CI 0.851-0.899) after adding Storyteller (ΔAUC 0.019, 95% CI 0.008‐0.030; *P*<.001). At the frozen threshold, the full model achieved sensitivity of 0.874 (95% CI 0.818-0.930), specificity of 0.746 (95% CI 0.725‐0.767), positive predictive value of 0.227 (95% CI 0.210‐0.246), and negative predictive value of 0.986 (95% CI 0.980‐0.992). The Brier score was 0.060, with a calibration intercept of 0.023 and a slope of 0.870, suggesting acceptable calibration with overfitting.

**Conclusions:**

Speech measures provided incremental information beyond Self-ECog12 and demographics, with and without additional subjective memory measures. These findings support further evaluation of multimodal remote assessment as a scalable screening or enrichment approach for identifying individuals who may benefit from clinical evaluation. Because the outcome was a participant-reported prior diagnosis rather than an independently adjudicated current diagnosis, validation against clinically adjudicated outcomes is necessary before diagnostic use.

## Introduction

Dementia is characterized by progressive cognitive and functional impairment that can ultimately compromise independent daily living. Alzheimer disease (AD), the most common cause of dementia, is associated with amyloid beta and tau pathology that develops well before the dementia stage [1]. With disease-modifying therapies now available for early AD [2], identifying people with mild cognitive impairment (MCI) or other early cognitive changes who may warrant further evaluation has become increasingly important. Approaches that can be administered remotely and at scale could help extend this identification beyond conventional clinic-based assessments [3,4].

Clinical characterization of MCI and dementia commonly incorporates formal neuropsychological assessment [5]. Such testing, however, requires trained personnel and substantial participant time and provides a structured assessment of performance rather than a direct measure of cognitive functioning in everyday life [6]. Remote assessment offers complementary ways to capture both perceived cognitive change and objectively observed cognitive behavior. The Everyday Cognition (ECog) scale was developed to assess changes in cognitively mediated everyday functioning relative to earlier functioning (specifically, 10 y previously) and can be completed by either the participant or an informant [7]. Self- and informant assessments may differ, particularly as awareness of cognitive difficulties declines with impairment [7,8], and shortened ECog measures have been evaluated for identifying cognitive impairment [9].

Speech provides a different source of information because it can capture cognitive performance during a task rather than relying on a participant’s perception of change. Automated story-recall approaches analyze spoken retellings to quantify features of recall and language without requiring manual scoring. Prior studies have shown that these measures are associated with early cognitive impairment and AD-related biomarker status across supervised and remote settings [10-15]. Storyteller has also been evaluated in the Alzheimer’s Disease Neuroimaging Initiative 4 (ADNI4) Remote Digital cohort: in 426 remotely assessed participants, speech-derived measures discriminated those reporting MCI, AD, or dementia from those without such a diagnosis [16]. Thus, prior work supports the feasibility of both subjective cognitive assessment and automated speech assessment outside conventional in-person testing.

What remains less clear is whether these 2 forms of remote assessment capture sufficiently different information to improve discrimination when used together. This is particularly important when the subjective component is already strong: the self-reported 12-item Everyday Cognition (Self-ECog12) scale captures perceived change across everyday cognitive functions, while additional questions can capture current memory/thinking concerns and perceived decline over a more recent period. ADNI4 provides an opportunity to examine this question because Self-ECog12 and Storyteller are collected remotely in the same cohort [17,18]. Previous studies have demonstrated discrimination using the remotely administered 12-item Everyday Cognition (ECog12) scale [19], and an Alzheimer’s Disease Neuroimaging Initiative (ADNI) analysis showed that incorporating questions about more recent concerns about memory or thinking improved discrimination beyond Self-ECog12 alone [20].

Accordingly, the present study asked whether automated speech recall provides additional discriminative information beyond remote questionnaire assessment, and whether that contribution persists when subjective memory questions that may be influenced by awareness of a prior diagnosis are excluded. We therefore evaluated the incremental contribution of Storyteller after Self-ECog12 and demographics alone and after a stronger subjective model that additionally incorporated current memory/thinking concerns and perceived recent memory decline. We hypothesized that (1) higher Self-ECog12 scores would be associated with greater odds of a prior cognitive impairment diagnosis in keeping with Manjavong et al [20] and (2) adding Storyteller measures would significantly improve discrimination of diagnostic status beyond questionnaire and demographic measures.

## Methods

### Participants and Data Sources

The ADNI is an ongoing multicenter longitudinal study established to develop and validate biomarkers and clinical measures for AD and related disorders [21]. ADNI4, the current phase of the initiative, expands participant recruitment and assessment through digital technologies, including remote cognitive screening and longitudinal monitoring. One component of ADNI4 is the Remote Digital cohort, in which participants complete questionnaires and smartphone-based cognitive assessments outside the traditional clinical setting. The present study used baseline data from this Remote Digital cohort, including the Remote Cohort and Storyteller tables distributed through the Laboratory for Neuro Imaging (LONI) platform. Participants in this study were drawn from a single source: the ADNI4 Remote Digital cohort, which comprises a Digital Screening study arm and a Remote Longitudinal Monitoring arm of in-clinic ADNI4 participants completing assessments outside the clinical setting [17,18]. Both arms are linked by a shared participant identifier (ADNIOnlineID) and complete the same battery of remote instruments at defined time points (month 0, month 6, month 12, and so on) [17,18]. This cross-sectional analysis used baseline (month 0) assessments collected between June 2023 and July 2025 from the ADNI4 Remote Digital cohort. Data were obtained from the Remote Cohort and Storyteller tables distributed through the LONI platform [13].

### Inclusion Criteria

Participants were included if they (1) had a baseline (month 0) Remote Cohort screening record; (2) had complete baseline data on the Self-ECog12, the 2 related self-reported memory questions, demographics, and all 5 Storyteller speech-recall composites; and (3) reported a prior diagnosis from a health care professional on at least 1 of 3 screening items, or reported no prior diagnosis on all 3 screening items.

### Ethical Considerations

ADNI4 study procedures, including the Remote Digital cohort protocol and informed consent materials, were reviewed and approved by the applicable institutional review boards (University of California, San Francisco; ATRI-011), and participants provided informed consent electronically before participation [17,18]. The original consent permits the collected information to be shared with other researchers for future research without obtaining additional permission from participants. The present study was a secondary analysis of previously collected, deidentified ADNI data obtained through the LONI data repository; no additional participant contact or consent was undertaken for this analysis. ADNI4 Remote Digital participants were not compensated for participation in the digital study. The data analyzed for this study were deidentified and accessed under ADNI’s controlled-access data-sharing procedures. No images or other materials in this manuscript or its supplementary materials permit identification of individual participants.

### Measurement

#### ECog Scale

This study used the baseline, Self-ECog12, a validated short form of the original 39-item ECog that samples all 6 ECog cognitive domains with 2 items each [7,9]. Each item used a 4-point response scale (1-4), with an additional “don’t know/not applicable” response coded as 9 and treated as missing. The Self-ECog12 score was calculated as the arithmetic mean of available item responses, consistent with prior ADNI analyses of the ECog12 [20]. A code audit confirmed this implementation; because the locally available ADNI documentation did not specify a validated minimum number of completed items, no alternative scoring rule was imposed. A parallel study partner–reported version (SP-ECog12) exists in the same dataset; analyses reported here used the self-reported version.

#### Additional Self-Report Memory Questions

In addition to Self-ECog12, participants completed 2 separate subjective cognitive questions. The first asked, “Are you concerned that you have a memory or other thinking problem?” [20]. The second asked whether the participant perceived that their memory had declined substantially compared with 1 year earlier. These questions are not components of Self-ECog12. While Self-ECog12 assesses perceived change across multiple domains of everyday cognitive functioning relative to approximately 10 years earlier, the additional questions capture current memory/thinking concern and more recent perceived memory change, respectively. Responses were coded as binary variables (yes=1, no=0); “don’t know” and “prefer not to say” responses were treated as missing. The current memory/thinking concern item was included because prior ADNI work showed that it improved discrimination when added to self-reported ECog12 [20]; the 1-year decline item provided a complementary measure of more recent perceived memory change.

#### Speech-Based Story Recall Task

Participants also completed Storyteller, a smartphone-administered, speech-based cognitive battery incorporating the Automated Story Recall Task (ASRT) [10-12]. The battery presents 2 short stories for immediate retelling (STORY1IMM, STORY2IMM), a category fluency distractor task, and a delayed retelling of the first story (STORY1DEL). Recall accuracy is scored automatically using G-match, a cosine-similarity metric (scaled 0‐100) between the semantic embeddings of the source story and the participant’s spoken retelling [10]. Neuropsychological measures of episodic memory and related cognitive domains have demonstrated diagnostic utility in AD [22]. Story-recall performance is impaired in AD and is sensitive to delay duration when comparing individuals with probable AD, vascular dementia, and normal cognition [23]. Five prespecified speech-derived composite measures were extracted from the Storyteller task (Table 1).

**Table 1.** Storyteller speech-derived measures.

Feature	Source retellings	Aggregation	Definition	Units
Immediate G-match	STORY1IMM+STORY2IMM	Mean	Semantic similarity between source story and immediate spoken recall	0‐100
Delayed G-match	STORY1DEL	Single delayed recall	Semantic similarity between source story and delayed spoken recall	0‐100
Mean word count	STORY1IMM, STORY2IMM, and STORY1DEL	Mean	Number of spoken words produced per recall	Words
Mean idea density	STORY1IMM, STORY2IMM, and STORY1DEL	Mean	Information units per word	Ratio
Mean speech rate	STORY1IMM, STORY2IMM, and STORY1DEL	Mean	Speaking rate across recalls	Words/second

#### Cognitive Impairment Diagnosis

For this study, “diagnosis” refers to a participant-reported prior diagnosis made by a health care professional; diagnoses were not independently adjudicated as part of the Remote Digital assessment. Diagnostic status was determined from 3 baseline screening questions asking whether a health care professional had ever informed the participant that they had (1) MCI or a memory problem, (2) AD, or (3) dementia. Participants answering “yes” to one or more questions were classified as having a cognitive impairment diagnosis, whereas participants answering “no” to all 3 were classified as having no reported diagnosis. Participants with only “don’t know” or “prefer not to say” responses were excluded. Throughout the remainder of the manuscript, “diagnosis” and “diagnostic status” refer to this participant-reported prior health care professional diagnosis [20].

#### Demographic Information

Demographic covariates included age at baseline, gender, coded educational attainment, and a binary indicator for White race. Education was represented using the ADNI Remote Digital coded ordinal education variable reflecting highest educational attainment. Gender was modeled categorically with indicator variables, with women as the reference category; the small other and prefer not to say groups were combined as other/unknown. Sensitivity analyses modeling education as categorical indicators produced essentially identical results.

### Statistical Analysis

#### Primary Analysis Cohort

The primary analyses were conducted using the complete-case ADNI4 Remote Digital cohort, preserving the observed diagnosis prevalence. Missing data were characterized in the full baseline cohort before complete-case exclusion. Little’s test was used to evaluate whether missing continuous analysis variables were consistent with missing completely at random (MCAR). To assess potential selection associated with complete-case inclusion, participants retained in the analytic cohort were compared with those excluded because of missing data using available demographic, questionnaire, and diagnostic-status variables. Patterns of Storyteller missingness were additionally examined against the source Storyteller records. Multiple imputation was considered after characterization of the missing-data pattern and mechanism. The complete-case cohort comprised 7268 participants, including 574 (7.9%) with a cognitive impairment diagnosis. Participants were randomly divided into training 5451 (75%) and independent test 1817 (25%) cohorts using stratified sampling to preserve diagnosis prevalence in both datasets. The independent test cohort comprised 1817 participants, including 143 diagnosed participants. All model development and threshold optimization were performed exclusively within the training cohort.

#### Descriptive Analysis

Descriptive statistics were calculated for the complete-case cohort overall and by diagnostic status. Continuous variables are presented as mean (SD) and compared using 2-sided Welch *t* tests. Categorical variables are presented as counts (percentages) and compared using chi-square tests. To assess potential selection bias, characteristics of participants included in the complete-case cohort were compared with those excluded because of missing data.

#### Discriminative Ability of ECog, Demographics, Subjective Memory, and Speech Features

Four primary logistic regression models and 1 parallel model (Model 2S) were developed to evaluate the incremental contribution of different predictor groups. Model 1 included the Self-ECog12 score alone. Model 2 additionally incorporated demographic variables (age, gender, race, and education). To evaluate the incremental contribution of speech without the 2 additional subjective memory measures, a parallel Model 2S added the 5 Storyteller measures (Immediate G-match [mean], Delayed G-match, mean word count, mean idea density, and mean speech rate) directly to Model 2. Model 3 added the 2 subjective memory variables assessing current memory/thinking concerns and perceived substantial memory decline during the previous year to Model 2. Model 4, the full model, added the 5 prespecified Storyteller speech-derived measures to Model 3. A fifth, minimal model evaluated the speech-derived variables alone.

Continuous predictors were standardized before model fitting. Gender was represented as a categorical variable using dummy variables, with women as the reference category; indicator variables were not standardized. Model performance was evaluated on the independent held-out test cohort using the area under the receiver operating characteristic curve (AUC). Because the prevalence of the diagnosis was low, average precision (area under the precision-recall curve) was also examined for incremental Storyteller comparisons. The operating threshold for binary classification was selected using 5-fold out-of-fold predictions generated within the training cohort by maximizing the Youden index, and it was subsequently frozen before evaluation of the independent test cohort. Performance at this threshold was summarized using sensitivity, specificity, positive predictive value (PPV), negative predictive value (NPV), and overall accuracy. Integrated discrimination improvement and category-free net reclassification improvement for Model 3 vs Model 4 were examined as exploratory reclassification analyses and are reported in Table S4 and S5 in Multimedia Appendix 1.

#### Model Comparison and Calibration

Incremental improvements in discrimination between nested models were evaluated using the paired DeLong test [24]. The 95% CIs for AUC and threshold-dependent performance metrics were estimated using bootstrap resampling. Differences in average precision were evaluated using paired outcome-stratified bootstrap resampling. Model calibration was assessed using calibration plots, the Brier score, the calibration intercept, and the calibration slope. Logistic regression coefficients are reported as odds ratios with corresponding 95% CIs.

As secondary robustness checks distinct from the primary natural-prevalence analysis, sensitivity analyses included (1) random 1:1 undersampling, (2) demographic matching, (3) restriction of the positive outcome to participants reporting AD or dementia, (4) exclusion of race from the prediction models, (5) modeling education categorically rather than as a single ordinal predictor, and (6) comparison of included vs excluded participants to evaluate potential selection bias. Statistical significance was defined as *P*<.05. All analyses were performed in Python using pandas, scikit-learn, SciPy, and statsmodels.

## Results

### Participants and Demographic and Clinical Characteristics

A total of 7268 ADNI4 Remote Digital participants had complete baseline data on Self-ECog12, 2 additional subjective memory questions, demographic variables, and all 5 Storyteller speech-recall measures and constituted the primary analytic cohort. Among these participants, 574 (7.9%) had a cognitive impairment diagnosis and 6694 (92.1%) did not. Table 2 summarizes demographic, questionnaire, and speech-recall characteristics by diagnostic status. Diagnosed participants were older, had lower educational attainment, and had higher Self-ECog12 scores than participants without a diagnosis. They were also substantially more likely to report current memory/thinking concerns and to perceive substantial memory decline during the previous year. Across all 5 Storyteller measures, diagnosed participants demonstrated poorer recall and speech performance, including lower Immediate G-match (mean) and Delayed G-match scores, fewer spoken words, lower idea density, and slower speech rates (all *P*<.001).

**Table 2.** Participant characteristics by cognitive impairment diagnostic status.^a^

Characteristics	Prior diagnosis (n=574)	No prior diagnosis (n=6694)	Statistics	*P* value
Age (y), mean (SD)	69.31 (8.05)	66.41 (7.25)	8.35 (655.3)^b^	<.001
Gender, n (%)			90.73 (3)^c^	<.001
Men	194 (33.8)	1223 (18.3)		
Women	376 (65.5)	5459 (81.6)		
Other	4 (0.7)	10 (0.1)		
Prefer not to say	0 (0)	2 (0.03)		
Education category^d^, mean (SD)	15.77 (2.31)	16.47 (2.2)	−6.96 (665.9)^b^	<.001
Self-ECog12^e^, mean (SD)	2.09 (0.67)	1.42 (0.4)	23.65 (610.9)^b^	<.001
Memory concern, n (%)	557 (97.0)	3443 (51.4)	444.30 (1)^c^	<.001
Perceived 1-year decline, n (%)	371 (64.6)	1236 (18.5)	654.37 (1)^c^	<.001
Speech-recall features (Storyteller), mean (SD)				<.001
Immediate G-match (mean)^f^	57.23 (15.56)	69.58 (12.25)	−18.53 (635.4)^b^	
Delayed G-match	47.88 (18.57)	62.51 (14.73)	−18.40 (636.3)^b^	
Mean word count	62.07 (31.80)	83.81 (29.59)	−15.80 (660.9)^b^	
Mean idea density	0.41 (0.06)	0.43 (0.04)	−6.07 (606.6)^b^	
Mean speech rate (words/second)	1.86 (0.48)	2.11 (0.40)	−12.04 (645.5)^b^	

a Diagnostic status reflects the participant’s report of a prior health care professional diagnosis, as defined in the Methods section.

b *t* test (*df*).

c *χ*2 (*df*).

d Education category is a coded ordinal scale of highest educational attainment, not literal years; higher values indicate greater attainment.

e ECog12: 12-item Everyday Cognition scale.

f G-match: a scaled (0‐100) cosine similarity metric between the embeddings of the source story and the participant’s spoken retelling.

Participant selection began with 12,075 ADNI4 Remote Digital participants at baseline (Figure 1). After sequential exclusion of 501 participants with a missing or ambiguous outcome, 236 without a usable Self-ECog12 score, 319 without complete demographic or subjective-memory data, and 3751 without all 5 Storyteller composites, 7268 participants remained in the final complete-case cohort (574 diagnosed and 6694 without a diagnosis; diagnosis prevalence, 7.9%).

**Figure 1.**
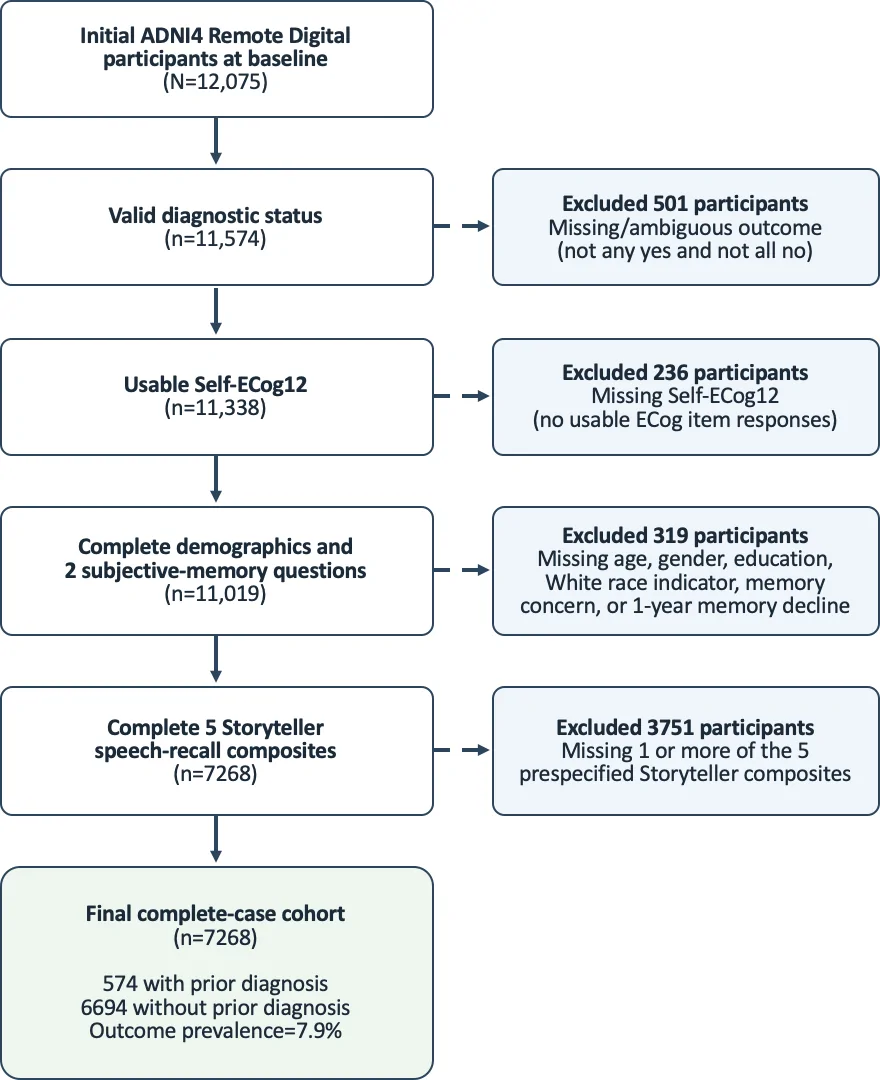
Participant flow and sequential exclusions leading to the final complete-case analytic cohort. ADNI4: Alzheimer's Disease Neuroimaging Initiative 4; Self-ECog12:self-reported 12-item Everyday Cognition.

In the full baseline cohort of 12,075 participants, 4807 (39.8%) had at least one missing primary-analysis variable. Missingness was concentrated in Storyteller: 4267 (35.3%) participants lacked all 5 baseline Storyteller measures, whereas only 67 (0.6%) had partially missing Storyteller measures. Of the 4267 participants lacking all 5 measures, 4263 had no baseline Storyteller record in the available source data; 504 of these had Storyteller data at a subsequent remote visit, whereas 3759 had no Storyteller record at any available visit. Little’s MCAR test rejected the hypothesis that the continuous analysis variables were MCAR (*χ*²_20_=1770.8; *P*<.001).

### Association Between ECog and Diagnostic Status

Hypothesis 1 predicted that higher Self-ECog12 scores would be associated with greater odds of a cognitive impairment diagnosis. In the multivariable logistic regression model, each 1-SD increase in Self-ECog12 score was associated with a nearly 2-fold increase in the odds of diagnosis (odds ratio 1.945, 95% CI 1.768‐2.140; *P*<.001). Older age, current memory/thinking concern, perceived substantial memory decline over the previous year, lower Delayed G-match, lower mean Immediate G-match, and slower speech rate were also associated with the outcome after adjustment for the other predictors. In contrast, education category, mean word count, and mean idea density were not independently associated with diagnosis after adjustment for the other predictors (Table 3).

**Table 3.** Multivariable logistic regression associations with prior cognitive impairment diagnosis among 7268 participants in the complete-case cohort.^a^

Predictor	Coefficient	OR^b^ (95% CI)	*P* value
Intercept	−5.789	0.003 (0.002‐0.005)	<.001
ECog-12^c^ mean score	0.665	1.945 (1.768‐2.140)	<.001
Age (y)	0.403	1.496 (1.353‐1.654)	<.001
Men (vs women)	0.642	1.900 (1.516‐2.383)	<.001
Other/unknown (vs women)	1.218	3.379 (0.894‐12.780)	.07
Education category	0.037	1.038 (0.939‐1.148)	.46
White race	0.576	1.779 (1.300‐2.435)	<.001
Memory/thinking concern	2.204	9.063 (5.453‐15.065)	<.001
Substantial memory decline vs 1 year ago	0.435	1.545 (1.230‐1.940)	<.001
Immediate G-match (mean)	−0.184	0.832 (0.703‐0.984)	.03
Delayed G-match	−0.366	0.694 (0.598‐0.804)	<.001
Mean word count	−0.006	0.994 (0.841‐1.175)	.95
Mean idea density	−0.014	0.986 (0.916‐1.062)	.71
Mean speech rate	−0.163	0.850 (0.769‐0.939)	.001

a Continuous predictors were standardized to 1 SD before model fitting; binary predictors remained coded as 0 and 1. Gender was modeled as a categorical predictor using dummy-variable encoding with women as the reference category. Coefficients and odds ratios were obtained from the unpenalized multivariable logistic regression model fitted to the complete-case cohort.

b OR: odds ratio.

c ECog12: 12-item Everyday Cognition scale.

Modeling education as categorical rather than as a single ordinal predictor produced nearly identical discrimination (Model 3: AUC 0.856, 95% CI 0.830-0.882; Model 4: AUC 0.877, 95% CI 0.853-0.900; ΔAUC 0.021, 95% CI 0.010-0.032; paired DeLong *P*<.001), indicating that the estimated incremental contribution of Storyteller measures was not materially affected by the specification of the education variable (Table S7 in Multimedia Appendix 1).

### Discriminative Performance of Nested Models

Discrimination in the independent natural-prevalence test cohort (comprising 1817 participants, including 143 cases) is summarized in Table 4. Self-ECog12 plus demographics achieved an AUC of 0.827 (95% CI 0.792-0.859). Adding Storyteller directly to this model, without the 2 additional subjective memory measures, increased the AUC to 0.853 (95% CI 0.821-0.883). Adding the subjective memory measures produced a Model 3 (AUC 0.857, 95% CI 0.830-0.882), and the complete Model 4 achieved the highest discrimination (AUC 0.875, 95% CI 0.851‐0.899; Figure 2).

**Table 4.** Discriminative performance of nested logistic regression models in the independent natural-prevalence test cohort (N=1817; 143 cases).

Model	Features	AUC^a^ (95% CI)
Model 1: ECog-12^b^	1	0.792 (0.755‐0.828)
Model 2: +demographics	6	0.827 (0.792‐0.859)
Model 2S: +Storyteller speech	11	0.853 (0.821‐0.883)
Model 3: +subjective memory	8	0.857 (0.830‐0.882)
Model 4: +Storyteller speech	13	0.875 (0.851‐0.899)
Speech-only model	5	0.751 (0.707‐0.790)

a AUC: area under the receiver operating characteristic curve.

b Self-ECog12: self-reported 12-item Everyday Cognition scale.

**Figure 2.**
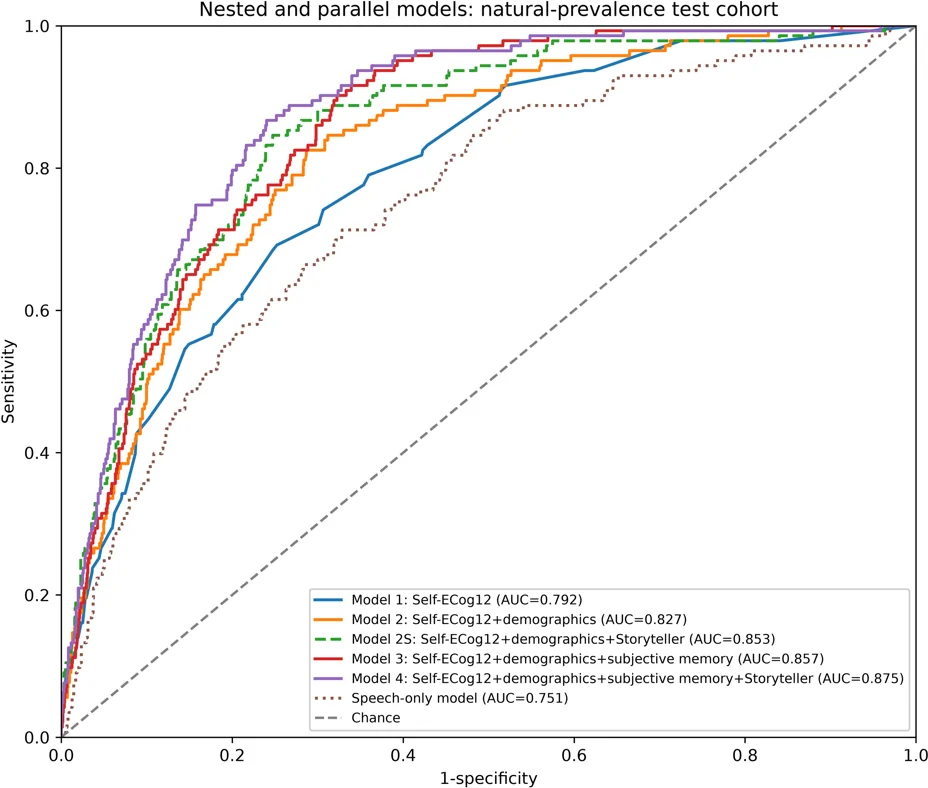
Receiver operating characteristic curves for the primary and parallel incremental logistic regression models and the speech-only model in the independent natural-prevalence test cohort (comprising 1817 participants, including 143 cases). Model 1 included Self-ECog12 alone; Model 2 additionally included demographic variables; Model 2S added the 5 Storyteller measures directly to Model 2 without including the 2 subjective memory measures; Model 3 additionally included the 2 subjective memory measures; and Model 4 additionally included the 5 Storyteller speech-recall measures. The dashed diagonal represents chance-level discrimination. AUC: area under the receiver operating characteristic curve; ECog12: 12-item Everyday Cognition scale.

### Incremental Value of Storyteller Speech-Recall Measures

Storyteller improved discrimination under both questionnaire baselines. When the 2 additional subjective memory measures were excluded, adding Storyteller to Self-ECog12 and demographics increased AUC from 0.827 (95% CI 0.792-0.859) to 0.853 (95% CI 0.821-0.883; ΔAUC 0.026, 95% CI 0.009‐0.043; paired DeLong *P*=.002). Average precision increased from 0.312 (95% CI 0.254-0.385) to 0.363 (95% CI 0.299-0.438; Δ 0.050, 95% CI 0.020‐0.085), compared with a diagnosis prevalence of 0.079. Thus, the incremental contribution of speech did not depend on inclusion of the 2 diagnosis-proximal subjective memory measures. When the subjective memory measures were included, adding Storyteller increased AUC from 0.857 (95% CI 0.830‐0.882) to 0.875 (95% CI 0.851‐0.899), corresponding to ΔAUC 0.019 (95% CI 0.008‐0.030; paired DeLong *P*<.001; Table 5).

**Table 5.** Pairwise comparisons of discrimination between nested models in the independent natural-prevalence test cohort.

Comparison	AUC^a^ A	AUC B	ΔAUC (95% CI)^b^	Paired DeLong *P* value^c^
Model 2 vs Model 2S: +Storyteller without subjective memory	0.827	0.853	0.026 (0.009‐0.043)	.002
Model 3: +subjective memory vs Model 4: +Storyteller speech	0.857	0.875	0.019 (0.008‐0.030)	<.001
Model 1: ECog-12^d^ vs Model 4: +Storyteller speech	0.792	0.875	0.084 (0.058‐0.110)	<.001

a AUC: area under the receiver operating characteristic curve.

b AUC and ΔAUC 95% CIs were estimated using 2000 outcome-stratified bootstrap resamples of the independent test cohort.

c *P* values were obtained using paired DeLong tests because model predictions were evaluated in the same participants.

d ECog12: 12-item Everyday Cognition scale.

### Threshold-Dependent Screening Performance

Using the threshold selected from 5-fold out-of-fold predictions during model development and frozen before test evaluation (probability threshold=0.066), the final model achieved sensitivity of 0.874 (95% CI 0.818‐0.930), specificity of 0.746 (95% CI 0.725‐0.767), PPV of 0.227 (95% CI 0.210‐0.246), NPV of 0.986 (95% CI 0.980‐0.992), and overall accuracy of 0.756 (95% CI 0.737‐0.775; Table S6 in Multimedia Appendix 1).

### Model Calibration

Calibration analysis demonstrated good agreement between predicted probabilities and observed event rates (Figure 3). The final model achieved a Brier score of 0.060, a calibration intercept of 0.023, and a calibration slope of 0.870, indicating little systematic calibration bias with some attenuation of predicted risk. Observed event rates were generally close to predicted probabilities across the range represented in the test cohort (Figure 3).

**Figure 3.**
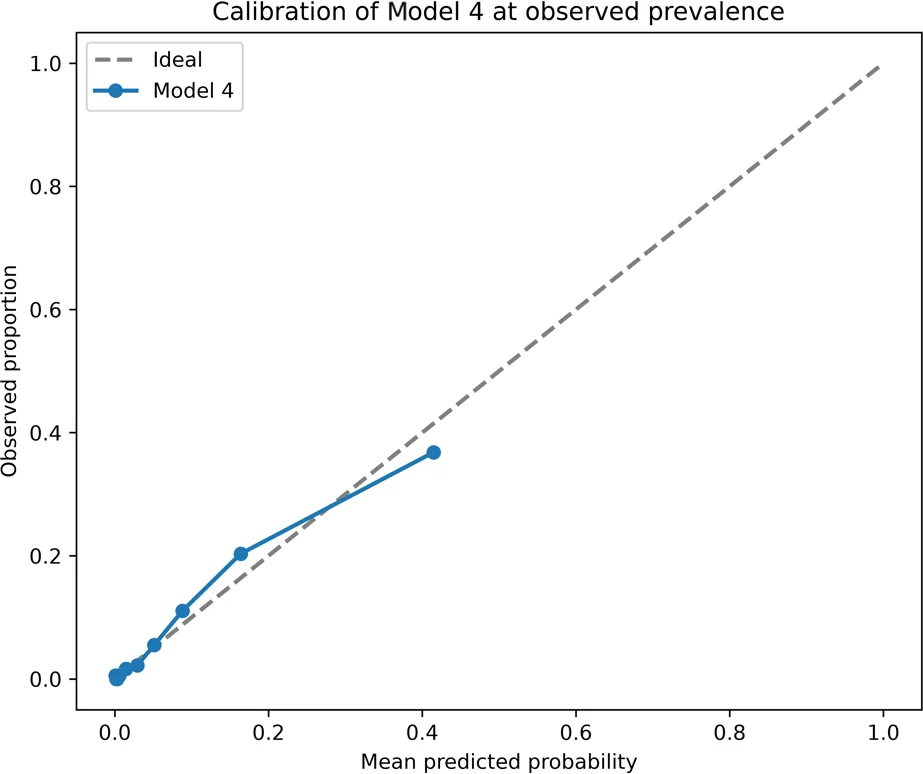
Calibration of the final questionnaire-plus-speech model in the independent natural-prevalence test cohort. The solid line and points show the observed cognitive-impairment proportions across groups of predicted probabilities; the dashed diagonal represents perfect calibration. The final model had a Brier score of 0.060, a calibration intercept of 0.023, and a calibration slope of 0.870.

### Feature Importance in the Full Model

Shapley additive explanations (SHAP) analyses were performed to characterize each predictor’s contribution to the final model (Figure 4). Current memory/thinking concern demonstrated the greatest overall influence on model predictions, followed by Self-ECog12 score and age. Among the speech-derived variables, Delayed G-match contributed more strongly than Immediate G-match (mean), whereas speech rate showed a more modest but consistent contribution. SHAP rankings describe contributions within the fitted multivariable model rather than unique or independent effects; correlated predictors may share predictive information. Among the 5 Storyteller measures, the largest absolute pairwise Pearson correlation was 0.797, between Immediate G-match (mean) and Delayed G-match, and the maximum variance inflation factor in Model 4 was 3.828 (Table S1 and S2 in Multimedia Appendix 1), indicating correlation among recall measures without evidence of severe multicollinearity. Higher Self-ECog12 scores, memory concern, age, and perceived substantial memory decline shifted predictions toward a diagnosis, whereas better story-recall performance and faster speech rates shifted predictions toward no diagnosis.

**Figure 4.**
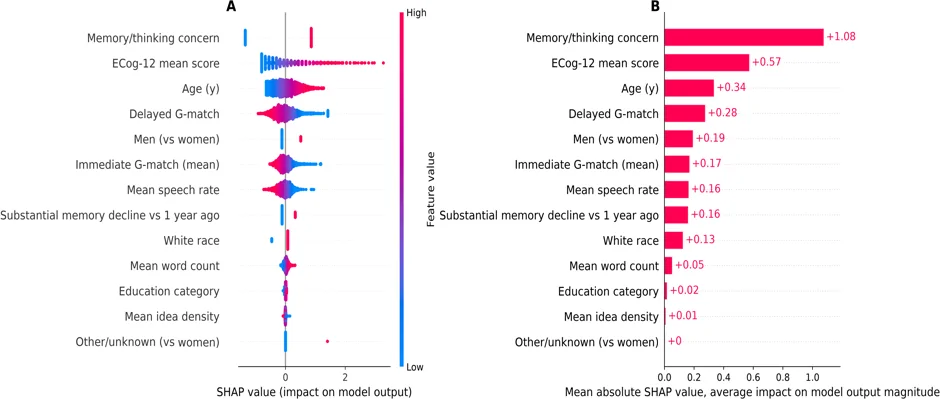
Shapley additive explanations (SHAP)–based feature contributions in the final multimodal model. (A) SHAP summary plot showing the distribution and direction of individual feature contributions; positive SHAP values shift predictions toward a diagnosis and negative values toward no diagnosis. Point color represents the observed feature value, from low (blue) to high (red). (B) Mean absolute SHAP values showing the average magnitude of each predictor’s contribution across participants. SHAP values describe the predictive contribution within the fitted model and do not represent causal or biological importance. ECog12: 12-item Everyday Cognition scale.

### Sensitivity Analyses

Sensitivity analyses supported the consistency of the principal findings across multiple analytic strategies (Table S7 in Multimedia Appendix 1). Excluding race produced essentially unchanged discrimination (Model 3: AUC 0.856, 95% CI 0.830-0.881; Model 4: AUC 0.873, 95% CI 0.848-0.896; ΔAUC 0.017, 95% CI 0.007-0.027; paired DeLong *P*=.002). Treating education categorically rather than as a single ordinal predictor likewise preserved the incremental speech contribution (Model 3: AUC 0.856, 95% CI 0.830-0.882; Model 4: AUC 0.877, 0.853-0.900; ΔAUC 0.021, 95% CI 0.010-0.032; paired DeLong *P*<.001). Comparisons of participants retained in vs excluded from the complete-case cohort showed negligible differences in age, gender, and cognitive-impairment prevalence, with greater differences in race, education, Self-ECog12, and the 2 subjective-memory measures (Table S2 in Multimedia Appendix 1). Restricting the outcome to AD or dementia yielded a similar absolute improvement (Model 3: AUC 0.930; Model 4: AUC 0.948; ΔAUC 0.018), although the paired comparison was not statistically significant (*P*=.08) among 167 cases. Across the 6 prespecified sensitivity analyses, the Model 3-to-Model 4 AUC increment ranged from 0.009 to 0.023.

## Discussion

### Principal Findings

The principal contribution of this study is to demonstrate that automated speech recall provides incremental information beyond remote questionnaire assessment under 2 complementary formulations. Storyteller improved discrimination both when added to Self-ECog12 and demographics alone and when added to the stronger subjective model that additionally included current memory/thinking concern and perceived recent decline. Thus, the contribution of objective speech-recall behavior did not depend on the diagnosis-proximal subjective memory measures and remained detectable after those measures were included. Importantly, this incremental value was demonstrated in a large remote cohort and evaluated in an independent test sample preserving the natural prevalence of previously reported cognitive impairment diagnoses. Together, these findings support multimodal remote assessment as a scalable screening or enrichment approach for identifying individuals who may benefit from further evaluation.

The definition of diagnosis is important when interpreting these findings. Diagnostic status was based on participants’ reports of a prior diagnosis made by a health care professional rather than on independent adjudication within this study. Knowledge of a previous diagnosis may influence subsequent responses to subjective measures such as Self-ECog12 and current memory concern, and people who seek or receive cognitive evaluation may differ systematically from those who do not. A parallel analysis that excluded the 2 additional subjective memory questions directly addressed part of this concern. Importantly, Storyteller continued to improve discrimination when added to Self-ECog12 and demographics without the 2 additional subjective memory measures. The results therefore demonstrate the recovery of previously reported diagnostic status using remote measures rather than a de novo clinical diagnosis. Importantly, the significant incremental contribution of Storyteller indicates that objective speech-recall behavior adds information beyond subjective self-report alone.

The contribution of subjective memory concern is consistent with prior ADNI work showing that adding a single memory-concern question improves the performance of self-reported ECog12 [20]. In the present study, memory/thinking concern again contributed strongly, although its proximity to diagnostic status warrants caution: participants who are aware of a previous diagnosis may be more likely to report concern, and concern itself may have prompted clinical evaluation. For this reason, Model 3 is best viewed as a deliberately strong subjective screening baseline. The complementary Model 2-to-Model 2S analysis shows that Storyteller also adds information when these diagnosis-proximal questions are omitted, while the Model 3-to-Model 4 analysis shows that speech remains informative even after they are included.

The incremental contribution of speech is particularly important in light of prior work establishing speech-based cognitive screening. Skirrow et al [16] demonstrated that Storyteller speech measures generalize to the ADNI4 Remote Digital cohort and discriminate among participant-reported MCI, AD, or dementia. More recently, Heitz et al [25] showed in 1003 older adults that automatically extracted speech features can predict cognitive-domain performance and identify individuals performing below normative cognitive thresholds, further supporting speech as a scalable digital marker of cognition [26]. The present study addresses a different question: whether speech contributes complementary information beyond remote questionnaire assessment across both a parsimonious baseline of Self-ECog12 plus demographics and a stronger baseline that additionally incorporates subjective memory concern and recent perceived decline.

The incremental speech contribution was also insensitive to 2 demographic modeling choices. Excluding race and modeling education categorically rather than as a single ordinal predictor produced essentially unchanged discrimination and incremental speech performance. Thus, the principal finding was not materially dependent on the inclusion of race or on assuming a linear effect for the coded education variable.

### Comparison With Prior Work

The most directly relevant prior study evaluated Self-ECog12 within ADNI and found that adding a single memory-concern question improved discrimination from approximately 0.70 to 0.79 [20]. Our findings are consistent with that result and extend it in an important direction: rather than asking whether another subjective question improves Self-ECog12, we tested whether objective speech-recall behavior contributes additional information both before and after diagnosis-proximal subjective memory measures are incorporated. The Self-ECog12 AUC observed here (0.792, 95% CI 0.755-0.828) was higher than the Self-ECog12 AUCs reported in a recent multicohort comparison of web-based and in-clinic ECog administration (0.709 and 0.747, respectively) [19]. This difference may reflect the nature of the outcome rather than a true difference in questionnaire performance: the previous study used clinician-adjudicated diagnoses, whereas diagnostic status in the present study was based on participant reports of a prior health care professional’s diagnosis. Nevertheless, the findings are consistent with that prior work in showing that ECog12 retains useful discriminative value when collected remotely [19].

Prior speech studies have established the feasibility and discriminative value of automated cognitive assessment, including remote Storyteller testing [10-16] and more recent large-scale work using speech as a proxy for cognitive performance [25]. Importantly, Skirrow et al [16] already demonstrated the performance of Storyteller in the ADNI4 Remote Digital population. The present analysis therefore does not seek to revalidate Storyteller as a stand-alone screener; rather, it demonstrates that Storyteller speech-recall measures provide statistically significant incremental discrimination beyond Self-ECog12 and demographics, with the incremental contribution persisting after subjective memory measures were incorporated. This complementarity supports the evaluation of multimodal remote assessments rather than treating questionnaire and speech measures as alternative screening approaches.

### Limitations

This study has several limitations. Most importantly, diagnostic status was based on participants’ reports of a prior health care professional’s diagnosis rather than independent clinical adjudication. Because diagnostic status was participant-reported and several important predictors were subjective questionnaire measures, some degree of circularity and ascertainment bias is possible. However, the incremental speech effect persisted when the 2 additional memory-concern measures most proximal to diagnostic awareness were excluded, reducing the likelihood that the principal speech finding depended on those predictors. Participants aware of a previous diagnosis may subsequently report greater everyday cognitive difficulty or memory concern, while individuals who seek clinical evaluation may differ systematically from those who do not. Access to health care is another source of potential ascertainment bias: participants with underlying cognitive impairment who had limited access to diagnostic evaluation could have been classified into the no-diagnosis group despite having clinically meaningful impairment. Accordingly, the reported discrimination should be interpreted as recovery of previously reported diagnostic status rather than as de novo diagnostic accuracy for underlying cognitive disease.

The positive outcome also combined “mild cognitive impairment or a memory problem,” AD, and dementia, spanning different severities and potentially different etiologies. Because the source ADNI item combines MCI and “memory problem,” those categories could not be separated. A sensitivity analysis restricted to participants reporting AD or dementia showed a similar incremental effect from Storyteller, although uncertainty was greater in this substantially smaller subgroup. This finding supports the consistency of the observed speech increment but requires confirmation in a larger clinically defined sample.

At the observed prevalence, the final model showed high NPV but modest PPV, supporting its interpretation as a potential screening or enrichment approach rather than a stand-alone diagnostic test. The study also used a single remote cohort at 1 time point, limiting inference about in-clinic performance, longitudinal change, or generalizability to other populations. The cohort was disproportionately White, which may further limit generalizability; however, exclusion of race produced essentially unchanged discrimination and speech-related incremental performance. Education was available as a coded ordinal attainment measure rather than literal years, but modeling it categorically also produced essentially unchanged results. Missing data present an additional limitation. The primary analysis was restricted to participants with complete data, and missingness was not consistent with MCAR. Missingness predominantly reflected absence of the entire baseline Storyteller assessment rather than sporadic missing speech measurements. Multiple imputation was considered but was not performed because rejection of MCAR does not establish the missing at random assumption required for conventional multiple imputation, and the available data did not identify why baseline Storyteller assessments were absent for most affected participants. Moreover, imputing an entirely unobserved speech modality primarily from the nonspeech predictors against which its incremental value was being evaluated would not directly assess the incremental information contained in observed speech. Differences between included and excluded participants therefore represent a potential source of selection bias and should be considered when generalizing these findings. In addition, depression was not available for adjustment despite its known association with subjective cognitive complaints [27]. Finally, the analysis used a deliberately simple logistic modeling framework and did not compare multiple machine-learning approaches, favoring interpretability and direct testing of incremental predictor groups over optimization of predictive performance. Despite these limitations, the ability of entirely remote questionnaire and speech measures to recover prior diagnostic status with good discrimination supports further evaluation of this approach in clinically adjudicated and more diverse populations.

A natural next step is to use Remote Digital assessments to determine whether longitudinal change in Self-ECog12 and Storyteller measures predicts subsequent cognitive decline or conversion to MCI or dementia. Such studies would establish whether the complementary signals observed here translate from the discrimination of prior diagnostic status into prospective screening and risk stratification.

### Conclusions

In this fully Remote Digital cohort, everyday-cognition questions combined with automated speech-recall measures discriminated participants with a prior cognitive impairment diagnosis with good accuracy. Storyteller speech measures added significant information beyond Self-ECog12 and demographics, and this contribution persisted after additional subjective memory measures were incorporated. These findings suggest that scalable, low-burden remote assessment can help identify individuals who may benefit from further clinical evaluation. Validation against independently adjudicated diagnoses will be an important next step toward prospective screening and referral use.

## Supplementary material

10.2196/108372 Multimedia Appendix 1 Additional sensitivity analyses.

## Abbreviations

AD: Alzheimer disease
ADNI: Alzheimer’s Disease Neuroimaging Initiative
ADNI4: Alzheimer’s Disease Neuroimaging Initiative 4
ASRT: Automated Story Recall Task
AUC: area under the receiver operating characteristic curve
ECog: Everyday Cognition
ECog12: 12-item Everyday Cognition
LONI: Laboratory for Neuro Imaging
MCAR: missing completely at random
MCI: mild cognitive impairment
NPV: negative predictive value
PPV: positive predictive value
Self-ECog12: self-reported 12-item Everyday Cognition
SHAP: Shapley additive explanations
