# Supplementary material for: Evaluating the Accuracy of Remote Questionnaire and Speech Measures in Detecting Cognitive Impairment in the ADNI4 Remote Digital Cohort: Cross-Sectional Study
Source: JMIR Neurotechnol. 2026 Nov 6;5:e108372. doi: 10.2196/108372 (PMC13749481; doi:10.2196/108372)
Supplement: Supplementary file 1 [file neuro-v5-e108372-s001.docx]

Multimedia Appendix 1: Additional sensitivity analyses

| **Variable** | **Immediate G-match (mean)** | **Delayed G-match** | **Mean word count** | **Mean idea density** | **Mean speech rate** |
| --- | --- | --- | --- | --- | --- |
| **Immediate G-match (mean)** | | | | | |
| *r* | 1 | 0.797 | 0.768 | 0.261 | 0.340 |
| *P* value | — | <.001 | <.001 | <.001 | <.001 |
| **Delayed G-match** | | | | | |
| *r* | 0.797 | 1 | 0.697 | 0.246 | 0.294 |
| *P* value | <.001 | — | <.001 | <.001 | <.001 |
| **Mean word count** | | | | | |
| *r* | 0.768 | 0.697 | 1 | 0.211 | 0.319 |
| *P* value | <.001 | <.001 | — | <.001 | <.001 |
| **Mean idea density** | | | | | |
| *r* | 0.261 | 0.246 | 0.211 | 1 | 0.146 |
| *P* value | <.001 | <.001 | <.001 | — | <.001 |
| **Mean speech rate** | | | | | |
| *r* | 0.340 | 0.294 | 0.319 | 0.146 | 1 |
| *P* value | <.001 | <.001 | <.001 | <.001 | — |

**Table S1. Correlation analysis (Pearson r and two-tailed P value) among Storyteller speech-derived measures.**

Pearson correlations were calculated using the complete-case analytic cohort. P values are two-tailed. *r*: Pearson correlation coefficient.

**Table S2.** Variance inflation factors for predictors in the final multivariable model.

| **Predictor/design-matrix column** | **VIF** |
| --- | --- |
| ECog-12 mean score | 1.728 |
| Age (years) | 1.077 |
| Male (vs Female) | 1.048 |
| Other/Unknown (vs Female) | 1.004 |
| Education category | 1.126 |
| White race | 1.072 |
| Memory/thinking concern | 1.461 |
| Substantial memory decline vs 1 year ago | 1.573 |
| Immediate G-match (mean) | 3.828 |
| Delayed G-match | 2.961 |
| Mean word count | 2.676 |
| Mean idea density | 1.093 |
| Mean speech rate | 1.185 |

The maximum variance inflation factor was 3.828, indicating no evidence of problematic multicollinearity.

**Table S3.** Precision-recall performance in the independent natural-prevalence test cohort.

| **Model** | **Average precision / PR-AUC (95% CI)** | **Difference vs Model 3** | **Test N (cases)** |
| --- | --- | --- | --- |
| Model 3 | 0.324 (0.271-0.395) | — | 1817 (143) |
| Model 4 | 0.379 (0.315-0.454) | 0.055 (0.017-0.087) | 1817 (143) |
| No-skill reference | 0.079 | — | 1817 (143) |

**Table S4.** Exploratory integrated discrimination improvement for Model 3 versus Model 4.

| **Metric** | **Model 3** | **Model 4** | **Difference** |
| --- | --- | --- | --- |
| Mean predicted probability, events | 0.2464 | 0.2715 | +0.0251 |
| Mean predicted probability, nonevents | 0.0645 | 0.0609 | -0.0037 |
| Discrimination slope | 0.1819 | 0.2107 | +0.0288 |
| IDI | — | — | 0.0288 (95% CI 0.0114–0.0458) |
| Bootstrap P value | — | — | 0.003 |

**Table S5.** Exploratory category-free net reclassification improvement for Model 3 versus Model 4.

| **Component** | **Count / proportion** | **NRI contribution** |
| --- | --- | --- |
| Events: moved up | 86 / 0.601 |  |
| Events: moved down | 57 / 0.399 |  |
| Events: unchanged | 0 / 0.000 |  |
| NRI event (event N=143) |  | 0.203 |
| Nonevents: moved down | 1126 / 0.673 |  |
| Nonevents: moved up | 548 / 0.327 |  |
| Nonevents: unchanged | 0 / 0.000 |  |
| NRI nonevent (nonevent N=1674) |  | 0.345 |
| NRI total |  | 0.548 |
| 95% CI |  | 0.378–0.715 |
| Bootstrap P value |  | <.001 |

**Table S6.** Threshold-dependent classification performance of the final model in the independent natural-prevalence test cohort.

| **Metric** | **Estimate (95% CI)** |
| --- | --- |
| Sensitivity | 0.874 (0.818-0.930) |
| Specificity | 0.746 (0.725-0.767) |
| PPV | 0.227 (0.210-0.246) |
| NPV | 0.986 (0.980-0.992) |
| Accuracy | 0.756 (0.737-0.775) |
| F1 score | 0.361 |
| Youden index | 0.620 |
| Frozen decision threshold | 0.066 |
| Confusion matrix (TN, FP, FN, TP) | 1249, 425, 18, 125 |

^a^The operating threshold (0.066) was selected by maximizing the Youden index using five-fold out-of-fold predictions in the training cohort and was frozen before evaluation in the independent test cohort. PPV: positive predictive value; NPV: negative predictive value; CI: confidence interval.

**Table S7.** Sensitivity analyses of the incremental discrimination associated with Storyteller speech-recall measures.

| **Analysis** | **N** | **Cases** | **Model 3 AUC** | **Model 4 AUC** | **Delta AUC** | **Paired DeLong P** |
| --- | --- | --- | --- | --- | --- | --- |
| Natural prevalence | 7268 | 574 | 0.857 | 0.875 | 0.019 | <.001 |
| Random 1:1 undersampling | 1148 | 574 | 0.870 | 0.879 | 0.009 | 0.356 |
| Demographic matched | 1136 | 568 | 0.827 | 0.850 | 0.023 | 0.037 |
| AD/dementia-only outcome | 6861 | 167 | 0.930 | 0.948 | 0.018 | 0.082 |
| Race excluded | 7268 | 574 | 0.856 | 0.873 | 0.017 | 0.002 |
| Education categorical | 7268 | 574 | 0.856 | 0.877 | 0.021 | <.001 |

**Figure S1. Precision-recall curves for the final subjective and multimodal models.** Precision-recall curves in the independent natural-prevalence test cohort for Model 3, which included Self-ECog12, demographic variables, and the two subjective memory measures, and Model 4, which additionally included the five Storyteller speech-recall measures. The no-skill reference corresponds to the observed diagnosis prevalence in the test cohort (0.079). PR-AUC: area under the precision-recall curve; ECog12: 12-item Everyday Cognition scale.
